# Supplementary material for: Research on reconfigurable topology layered equalization method based on maximum capacity utilization
Source: PLoS One. 2023 Dec 14;18(12):e0295425. doi: 10.1371/journal.pone.0295425 (PMC10721023; doi:10.1371/journal.pone.0295425)
Supplement: S2 File — (DOCX) [file pone.0295425.s002.docx]

**Supporting information**

<https://doi.org/10.17605/OSF.IO/5MSR8>

<https://osf.io/5msr8/files/osfstorage/6562b1d3f0b8e22801a9d794>
